# Tick Preventive Behaviors and Practices Adopted by Medical Students from Poland, Germany, and Thailand in Relation to Socio-Demographic Conditions and Their Knowledge of Ticks and Tick-Borne Diseases

**DOI:** 10.3390/insects11120863

**Published:** 2020-12-03

**Authors:** Alicja Buczek, Johanna Pilch, Weronika Buczek

**Affiliations:** Chair and Department of Biology and Parasitology, Faculty of Health Sciences, Medical University of Lublin, Radziwiłłowska 11 St., 20-080 Lublin, Poland; pilchjoa@gmail.com (J.P.); wera1301@gmail.com (W.B.)

**Keywords:** ticks, preventive behaviors, tick prevention practices, prophylaxis, tick-borne diseases, knowledge of tick-borne diseases

## Abstract

**Simple Summary:**

Ticks are arthropods with the highest importance for public health. The effects of their parasitism can be limited by application of various prophylaxis methods. This study compares preventive behaviors and practices against tick bites and knowledge of ticks and tick-borne diseases declared by medical students from two European countries (Poland and Germany) and an Asian country (Thailand). The study revealed differences in the use of various preventive methods and practices and in the knowledge of ticks and tick-borne diseases declared by the respondents. The most popular preventive methods employed by the Polish and German students included inspection of the body on return home and wearing protective clothes. The Thai students chose wearing protective clothes and preventive behavior in tick habitats. Tick repellents were used by the students less frequently. Approximately 7% of the Polish medical students and 22% of the German and Thai respondents did not use any means of prevention. The insufficient knowledge of ticks and tick-borne diseases declared by the medical students and the unsatisfactory use of preventive methods indicates the need for education focused on threats posed to humans by biological environmental factors. It is also essential to popularize tick prophylaxis methods among the public.

**Abstract:**

Given the high medical importance of ticks, we analyzed the most common preventive behaviors and practices adopted by medical students from Poland, Germany, and Thailand, and the level of their knowledge of ticks and tick-borne diseases. A survey consisting of 19 questions was conducted among 636 randomly selected students. The study showed that the Polish and German students preferred inspection of the body on their return home (86.9% and 63.5%, respectively) and wearing protective clothes (79.8% and 32.3%, respectively) as part of prophylaxis. The Thai students most often chose wearing protective clothes (54.7%) and preventive behavior in tick habitats (42.7%). Approximately 7% of the Polish medical students and as many as 22% of the German and Thai respondents did not use any means of prevention. Our analyses suggest that the use of preventive methods and respondents’ behaviors depend on socio-demographic factors and the level of health education. The insufficient practical implementation of tick prevention measures by the medical students suggests a need for verification of health education programs in schools as well as effective popularization and educational activities. It is also necessary to develop a public health protection strategy against the effects of tick bites.

## 1. Introduction

Ticks are one of the most important biological factors in the environment, posing a threat to human health. Approximately 10% of the circa 900 identified species are vectors of tick-borne pathogens, including agents of infectious diseases with considerable importance for public health [1,2]. In addition to the role of ticks in pathogen transmission, the components of their saliva cause skin lesions and systemic reactions in humans [3,4,5,6]. Neurotoxins secreted by tick salivary glands during feeding on the host may cause tick paralysis [7,8,9,10], while other components of saliva may trigger allergies to meat [11,12,13,14,15].

The increase in the tick occurrence range and abundance induced by climate and environmental changes raises the risk of human exposure to the bites of these arthropods and pathogen infections [16,17,18,19,20,21]. A measurable effect of this phenomenon is the dramatic increase in the incidence of human tick-borne diseases worldwide, including Lyme disease, rickettsiosis, Crimean Congo hemorrhagic fever, and tick-borne encephalitis [22,23,24,25,26,27,28,29,30].

Human efforts aimed at reduction of the number of ticks with chemical or biological methods have proved ineffective and unsatisfactory. This is related to e.g., the resistance to some acaricides developed by these arthropods [31,32], the toxicity of chemical substances to other organisms present in the same ecosystem [33,34,35], and the high costs of tick control treatments [36]. Tick control is also problematic due to the specific biological and physiological traits of these arthropods, i.e., the ability to increase the population size substantially within a short time [37], cover long distances while feeding on migrating hosts, and adapt to new habitat conditions [38,39].

Therefore, increasing attention is currently being focused on human personal protection against tick attacks and on popularization of tick prophylaxis and knowledge of the problem.

In this paper, we compared the preventive behaviors and practices against tick bites and the knowledge of ticks and tick-borne diseases declared by medical university students from different geographical and cultural regions, i.e., Poland, Germany, and Thailand. We also investigated the impact of various factors on their decisions in the choice of tick prophylaxis methods, which can be used for development of strategies for the prophylaxis of tick-borne diseases.

## 2. Materials and Methods

### 2.1. Participants

The survey respondents were medical students from three countries, i.e., Poland, Germany, and Thailand, who studied at two European universities in 2018–2019. They studied at the Medical University of Lublin (Lublin Province, southeast Poland) (Polish and Thai students assigned into groups 1 and 3, respectively) and at the University of Göttingen (Lower Saxony, northwest Germany) (German students, group 2). The survey was conducted among students who had biology classes during their University stay. The randomly selected respondents gave consent to participate in the survey. The involvement of the Polish, German, and Thai students in the study allowed a comparison of the use of various types of preventive methods in countries located in different regions of the world and differing in their economy and culture.

Group 1 with 99 respondents consisted of mainly third- and first-year Polish students at the Faculty of Medicine, who lived and were educated only in Poland.

Group 2 comprised 453 respondents, mainly fourth-, second-, and first-year dentistry students, who grew up and attended schools only in Germany.

Group 3 consisted of 84 first-to-sixth-year Thai students of medicine (mostly first-year students, but a majority of the respondents did not provide this information in the questionnaire), who studied at the Medical University of Lublin under the bilateral agreement between the governments of Poland and Thailand. The students had never been to Europe before they started studying. Before filling in the questionnaires, the Thai students had been in Poland for a short time. They also did not know the Polish language. Therefore, any impact of Polish media and newspapers on their views and answers to the survey questions was ruled out. The Thai students were asked to answer questions about preventive methods of protection against tick bites based on their experience gained in their country.

The number of the Thai respondents reflects the low number of Thai students enrolled in the Medical University of Lublin during the research period. A similar-sized group of respondents was randomly selected among Polish students, which prevented potential errors in the interpretation of results caused by the differences in the number of participants from the European and Asian countries. As shown by the statistical analyses, the higher number of German respondents did not affect the result of this comparative study.

### 2.2. Questionnaire

The anonymous questionnaires contained 19 questions in Polish, German, or English compiled in four subsets. The first subset required general information about the respondents, e.g., the country of origin and permanent place of residence (town/village), age, year of study, and previous education. The second subset included questions about tick attack events (single/repeated more than twice), the circumstances of tick attack (recreation/work in tick habitats, urban/rural area, recreational behavior, and the time of day of the tick attack on the respondent). There were also questions about symptoms observed after tick bites (local/systemic) and the use of drugs after tick infestations. The third part was focused on the preventive methods used by the respondents and the motivations for the choice. The questions about the preventive measures and practices addressed the use of repellents, wearing clothes protecting from tick bites and making ticks discernible (bright clothes, long trousers, foot-covering shoes, long-sleeved sweatshirts), avoidance of sitting on the grass in areas of potential tick occurrence, other methods for prevention of tick exposure, inspection of the body on return home, and the use of other prophylaxis methods. Several reasons for employing tick prophylaxis were proposed, e.g., the risk of tick-borne disease, fear of tick attacks and infection with pathogens, simplicity of prophylaxis methods, etc.

The fourth part of the questionnaire was focused on the sources of information about ticks and tick-borne diseases. The sources of information suggested in the questions included primary, secondary, and university education, television, popular science magazines, electronic media, family/friends, healthcare facilities, medical personnel, etc.

Various methods were used to obtain detailed information from the respondents, i.e., single choice questions (the first subset), multiple choice questions (the second, third, and fourth subsets), and semi-closed questions accompanying questions in all subsets. The semi-closed questions did not limit participants’ responses but allowed them to express their opinions on the topic fully. The answers from all the respondents were analyzed collectively and separately for the Polish, German, and Thai groups. This revealed the attitudes of the medical students, regardless of their demographic, educational, and cultural background, and helped to trace similarities and differences in the use of tick prophylaxis measures, preventive behaviors, and knowledge declared by the Polish, German, and Thai students. Moreover, we investigated whether the tick prophylaxis methods and respondents’ knowledge of ticks were correlated with the differences in their living conditions and education level.

### 2.3. Statistical Analysis

In the case of qualitative variables, the frequency and percentage of occurrence of each category were determined. The following parameters of descriptive statistics were used in the analysis of quantitative variables: arithmetic mean, standard deviation, median, minimum value, and maximum value.

Pearson’s Chi^2^ (χ^2^) test of independence was used to verify the statistical hypotheses. It is based on comparison of observed values with expected values (i.e., those assumed by the test when there is no relationship between the variables). If the difference between the observed and expected values is large (statistically significant), a correlation between the variables can be assumed. A significance level of *p* < 0.05 was adopted in the analysis.

The database and statistical analysis were based on the use of IBM SPSS Statistics (version 25) computer software.

## 3. Results

Approximately 52.6% of the 636 respondents reported a previous tick bite incident, with the largest number of the German students (59.2%) and a lower number of the Polish (46.5%) and Thai (23.8%) students. The differences between the three groups (chi^2^ = 37.234; *p* < 0.001) and between the Polish and German (chi^2^ = 4.835; *p* = 0.028), Polish and Thai (chi^2^ = 9.156; *p* = 0.002), and German and Thai (chi^2^ = 34.203; *p* < 0.001) students were statistically significant. The mean age of all respondents was 23.17 ± 3.44 years, and the mean age of the Polish, German, and Thai students was 20.86 ± 1.26, 23.82 ± 3.56, and 22.38 ± 3.21 years, respectively.

The greatest numbers of the Polish respondents were second-year students (69.7%), whereas first-year students accounted for a substantially lower percentage (24.2%) (Table 1). The fourth- (25.6%), second- (19.4%), and first-year (11.2%) students dominated in the group of the German respondents. The third- fifth-, and sixth-year students represented a lower percentage, i.e., 9.9%, 8.2%, and 2.6%, respectively. In the group of the Thai respondents, there were 77.4% of first-year students and only 9.5% of second-year, 3.6% of third-year, 7.1% of fourth-year, and 2.4% of sixth-year students. The large number of the Thai students living in Poland for a short period limits the possibility of an impact of the Polish community (e.g., television, colleagues) on their answers to some of the questions, e.g., their knowledge and sources of information about ticks and tick-borne diseases.

Females dominated in groups 1 and 2, accounting for over 69% of the respondents in each. The percentage of females and males in group 3 was similar, i.e., 48.2% and 49.4%, respectively. A majority of the Polish and German students, i.e., 73.7% and 79.2%, respectively, were city residents. 20.2% of the Thai respondents lived in cities and only 1.2% lived in rural areas. 78.3% of them did not specify their place of living at all (Table 1).

The series of questions of the methods for protection from tick bites was answered by 620 students. The answers differed statistically significantly in the three studied groups (chi^2^ = 85.355; *p* < 0.001). Inspection of the body on return home (63.1%) and wearing protective clothes (42.6%) were regarded as the most effective methods for prevention of tick attacks. Approximately 20.5% of the respondents declared preventive behavior such as avoidance of sitting on the grass in areas of potential tick occurrence or avoidance of contact with plants where these ectoparasites quest for their host. Over 19% of the respondents declared the use of repellents. As many as 20% of respondents did not take any tick prophylaxis measures (Table 2).

The most popular measures indicated by the Polish students included inspection of the body on return home (86.9%) and wearing protective clothes covering the body (79.8%). The respondents from this group also declared outdoor behavior that limits the possibility of contact with ticks, i.e., avoidance of sitting on the grass in potential tick habitats (37.4%). The German students most frequently protected themselves from tick bites by inspecting the body on return home (63.5%). Nearly half of this group of respondents declared wearing protective clothes (32.3%). In turn, the Thai students preferred wearing protective clothes (54.7%) and avoidance of sitting on the grass (42.7%) as preventive measures, whereas inspection of the body was declared less frequently (29.3%) (Table 2).

The use of repellents for personal protection against tick attacks was mostly declared by the Polish students (30.3%), whereas a nearly two-fold lower number of the German (17.7%) and Thai students (14.7%) were found to take this prophylaxis measure. The Polish students were also the least numerous group of respondents that did not use any preventive measure (7.1%). In comparison with the Polish group, a substantially higher percentage of the German and Thai respondents (22% in each group) declared that they did not take any protective measures against tick bites during outdoor activities in periods of tick seasonal activity (Table 2).

There were statistically significant differences in the choice of preventive methods and practices between the Polish and German (chi^2^ = 46.896; *p* < 0.001), Polish and Thai (chi^2^ = 34.280; *p* < 0.001), and German and Thai (chi^2^ = 50.253; *p* < 0.001) respondents. The females and males from the same country did not differ in their choice of methods of protection against tick bites (Table 3). In turn, we found statistically significant differences in the application of various tick prophylaxis methods between the females (chi^2^ = 56.476; *p* < 0.001) and males (chi^2^ = 29.564; *p* = 0.001) from different countries.

The most common reason for taking tick prophylaxis measures indicated by the Polish (83.8%) and German (33.6%) students was the risk of development of tick-borne diseases. Most of the students from Thailand did not answer the question. As declared by the respondents, the other motivations for protection against tick bites and preventive behavior in tick habitats were less important. More frequently than the other survey respondents, the German students underlined that prophylaxis of tick bites and tick-borne diseases is easy to implement and inexpensive (16.3%) (Table 2).

As many as 56.2% of the 636 students asked to assess their awareness of the risk posed by ticks and tick-borne pathogens to human health considered it sufficient, whereas approximately 11% declared an insufficient level of knowledge. Approximately 27.3% did not give a clear answer (Table 4).

The Polish (59.5%) and German (62.3%) students declared having sufficient knowledge of the threats posed by ticks and tick-borne pathogens to human health. No precise answer was given by ca. 31.3% of respondents in group 1 and 25.2% in group 2. In turn, a majority of the Thai students reported insufficient knowledge (35.7%) or did not have an opinion on this issue (34.5%). Only 17.9% in this group declared sufficient knowledge. The statistical test revealed statistically significant differences between the three groups of students (chi^2^ = 92.137; *p* < 0.001) (Table 4). The knowledge of ticks assessed by the respondents differed statistically significantly between the Polish and German students (chi^2^ = 79.246; *p* < 0.001) and between the Polish and Thai respondents (chi^2^ = 89.243; *p* < 0.001).

The largest number of respondents declared that they acquired the knowledge of ticks, tick-borne diseases, and tick prophylaxis mainly from electronic media (42.0%), university education (42.3%), and to a lesser extent from high school (32.8%) and television (20.8%).

Although the Polish, German, and Thai students indicated similar sources of information about ticks, their preferences in the selection of the sources differed between the three groups, as confirmed by the statistical analysis (chi^2^ = 88.838; *p* < 0.001). The Polish students most frequently indicated higher education and secondary school as the source of knowledge, i.e., 66.7% and 57.6%, respectively.

Almost half of these respondents indicated other sources, primarily television (29.3%) and electronic media (26.3%). In turn, fewer German students indicated higher education (34.6%) and secondary school (29.2%) as a source of information. They often mentioned other sources, i.e., family/friends (20.5%) and popular science magazines (20.5%).

In comparison with the European students, a larger number of the Thai respondents indicated school as the main source of health education, including higher education (54.9%), secondary school, and primary school (21.1% each). As shown by the survey results, information provided by TV was a more frequent source of knowledge for the Polish students than for their German (19.4%) and Thai (18.3%) peers. Electronic media had an insignificant role in students’ knowledge of ticks in the case of the German (7.7%) and Thai (11.3%) respondents, whereas family and friends were an infrequent source for the Polish (6.1%) and Thai (4.2%) students. In all groups, information from health care facilities provided by medical personnel was declared by very few students as a source of knowledge. The statistical analysis showed statistically significant differences between the Polish and German students in terms of the sources of information (chi^2^ = 63.679; *p* < 0.001) and no such differences between the Polish and Thai students (chi^2^ =11.965; *p* = 0.153).

The respondents from the three groups indicated the lower limb, abdomen, or head as the most frequent tick attachment sites. The statistical test did not reveal significant differences in the location of the tick bites between the Polish, German, and Thai students (chi^2^ = 14.359; *p* = 0.278).

Most of the German (52.6%) and Thai (60%) respondents were attacked by ticks more than once (Table 5). In turn, the Polish students usually reported single tick infestations (56.5%) and less frequent repeated bites (43.5%). In all groups, the students declared contact with ticks mainly during leisure activities (60% and 94.8% of the Thai and German respondents, respectively). As many as 35% of the Thai respondents reported tick bite incidents associated with occupational activities, which were sporadic in the Polish and German groups. The statistical test revealed differences in the circumstances of tick bites reported by the respondents from the three groups (chi^2^ = 73.347; *p* < 0.001) (Table 4) and between the Polish and German (chi^2^ = 19.654; *p* < 0.001), Polish and Thai (chi^2^ = 14.890; *p* < 0.001), and German and Thai students (chi^2^ = 50.942; *p* < 0.001).

The tick attacks were usually reported by the Polish and German respondents to have taken place in non-urban areas, i.e., 84.8% and 78.4%, respectively (Table 5). In contrast, the tick infestations reported by the Thai students were more frequent in urban areas. They exhibited a two-fold higher frequency (65%) than that indicated by the German respondents (29.5%) and over three-fold higher frequency than that reported by the Polish students (17.4%). The differences were statistically significant between the three groups (chi^2^ = 14.844; *p* < 0.001) (Table 5) and between the Polish and Thai (chi^2^ = 13.669; *p* < 0.001) and German and Thai (chi^2^ = 11.210; *p* < 0.001) groups. However, there were no statistically significant differences between the two European groups (chi^2^ = 2.241; *p* = 0.134).

In the Polish, German, and Thai groups, the most frequent infestations were reported to have occurred in the afternoon, i.e., 50.0%, 63.4%, and 35.0%, respectively (Table 5). Noteworthy is the high percentage of Thai students attacked by ticks not only in the afternoon but also in the evening and at night, i.e., 20% and 30%, respectively. As confirmed by the statistical analysis, there were significant differences in the time of day when the tick attacks occurred between all the three groups (chi^2^ = 69.665; *p* < 0.001) (Table 5). The time of tick bites also differed significantly between the Polish and German (chi^2^ = 23.870; *p* < 0.001), Polish and Thai (chi^2^ = 12.154; *p* = 0.032), and German and Thai (chi^2^ = 54.473; *p* < 0.001) students.

**Figure 1:**
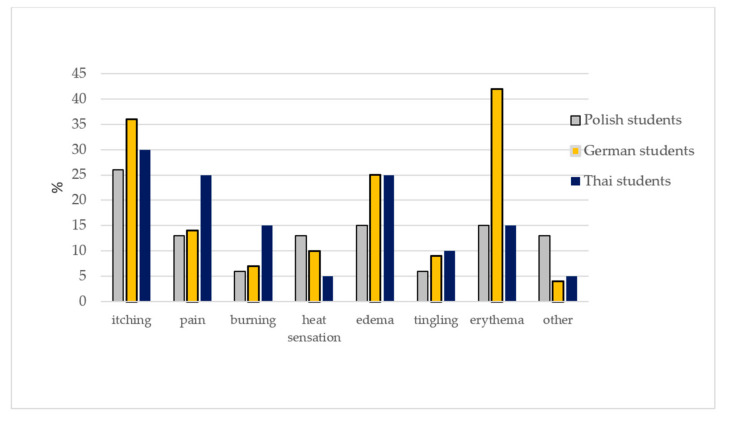
Symptoms at the tick bite site.

The respondents observed various symptoms at the bite site, e.g., itching, pain, erythema, swelling, heat sensation on the skin near the bite site, and a burning or tingling sensation (Figure 1). The differences between the Polish, German, and Thai students in the frequency of the local symptoms were not statistically significant (chi^2^ = 21.109; *p* = 0.098). The differences in the type of local symptoms of tick bites were statistically significant only between the Polish and German groups (chi^2^ = 14.422; *p* = 0.044).

The presence of systemic symptoms was indicated only by the Thai (35%) and German (4.1%) respondents, which accounted for 5.4% of students reporting tick bites. Statistically significant differences were found between the three groups (chi^2^ = 37.880; *p* < 0.001) and between the Polish and Thai (chi^2^ = 14.508; *p* < 0.001) and German and Thai (chi^2^ = 25.275; *p* < 0.001) students.

The German respondents most often reported malaise (72.7%) and fever (54.5%), whereas a lower percentage reported headaches and arthralgia (45.5% each). In turn, the Thai students mostly indicated fever (100%) and less often headaches (14.3%).

After tick infestations, drugs were most often administered to the German respondents (7.1%). Medicines were taken by only single subjects in the Polish and Thai groups, i.e., 4.3% and 5.0% of the 46 respondents that answered this question, respectively. The differences between the groups were not statistically significant.

## 4. Discussion

As shown in the present study, the residents of three countries: Poland, Germany, and Thailand are highly exposed to tick attacks. The differences in the number of individuals attacked by ticks and the frequency of tick infestations between the European and Asian respondents may be determined by various factors. These mainly include the presence and distribution of parasitic ticks in the three geographic regions and the socio-demographic characteristics of the surveyed groups of people, such as the country and place of residence and the behavior of the respondents (e.g., recreation in urban and non-urban areas, types of outdoor activities practiced by the respondents). The urban green areas and rural regions in Poland and Germany, i.e., the respondents’ place of residence, are habitats of *Ixodes ricinus* ticks, which most often attack humans in these two European countries and are the most important vectors of pathogens [40,41,42,43]. The frequency of tick bites in the group of students from Thailand, who mainly live in cities, was two-fold lower than that reported by their European peers. However, 60% of these respondents were infested by ticks at least twice. We did not have data on the natural environment in which the Thai students lived; hence, it was impossible to determine the species and abundance of ticks present in their living area. As reported by other authors [44,45], approximately 55 species representing 10 genera have been identified in Thailand so far, but their epidemiological role, related to transmission of tick-borne diseases, has been poorly explored.

Many pathogens have been detected in ticks collected in Central Europe [46,47,48] and South-East Asia [49,50,51,52,53,54]. These include *Borrelia burgdorferi* s.l. spirochetes and spotted fever group rickettsia species from the genera *Rickettsia, Anaplasma*, *Ehrlichia*, and *Babesia*. Additionally, ticks living in these regions are infected with arboviruses, which are highly pathogenic to humans, i.e., tick-borne encephalitis (TBE) virus in Europe [23,55,56] and Kyasanur Forest Disease (KFD) virus, Omsk Hemorrhagic Fever (OHF) virus, and Russian Spring Summer Encephalitis (RSSE) virus responsible for encephalitis and/or hemorrhagic syndromes in South-East Asia [57]. This proves the need to monitor the risk of tick attacks and take measures for protection of humans living in these regions.

The survey respondents used various preventive methods and practices that are part of two strategies for protection of humans against tick attacks. The first one consists of avoidance of ticks by wearing protective clothes preventing contact of these ectoparasites with the body and inspection of the body on return home. The other strategy involves application of repellents on the skin or clothes. Apart from the tick-borne encephalitis vaccine, there are no vaccines for the other tick-borne diseases [58].

The preferences for methods of protection against tick bites are similar in both European groups but differ from the preventive measures and practices declared by the Thai students. The Polish and German students most often focus on inspection of the body and, while staying outdoors, they wear protective clothes preventing ticks from contact with the skin. In turn, the Asian students pay considerable attention to their outdoor behavior and limit their contact with plants on which ticks may be present (e.g., avoidance of sitting on the grass, avoidance of places covered with shrubs and low vegetation). These measures are most frequently used by Czech students, as 50% of females and 38% of males declare that they protect themselves against ticks by wearing smooth and light-colored clothes and shoes as well as long sleeves [59]. In contrast to the findings reported by Nejezchlebovà et al. [59], the female and male students from the medical universities in Poland, Germany, and Thailand declared taking similar tick protective measures. In turn, the differences in the acceptance of preventive methods declared in the questionnaires by the females and males from both European countries and from the Asian country may be determined by various factors, e.g., the level of education, participants’ access to information about the harmful effects of tick parasitism, and mental and cultural characteristics of the individual groups of respondents.

Effective protection against tick bites can be provided by repellents. The most popular worldwide agents are formulations containing synthetic substances (e.g., deet, EBAAP [IR3535], icaridin [also known as picaridin], permethrin, AI3-37220, and SS220) or natural plant compounds (e.g., amyris essential oil, callicarpenal, carvacrol, common juniper essential oil, elemol, geraniol, and 2-undecanone) [60,61,62,63]. In comparison with the German and Thai respondents, the Polish students declared using repellents as a tick prophylaxis measure much more often. The differences between the groups of students in the choice of this method of prevention may be related to their awareness of both the effectiveness of repellents and the potential toxic effects of chemical substances on humans and the environment. Some importance may be ascribed to the mental differences between students from different cultural regions, their attitude to nature protection, and the manner and range of dissemination of advertising materials in the mass media (e.g., television, electronic media, and magazines).

The interest in repellents has been maintained among the inhabitants of southeastern Poland for many years. In 2005, the percentage of people who applied repellents to protect themselves against ticks was even higher (38%) than that reported in this study [64]. As declared by a group of students from another region of Poland located along the eastern border with Belarus, with a similar age and sex structure to the Polish and German groups of respondents, 15.6% always and 46.6% rarely use repellents, whereas 37.8% never use this tick prophylaxis method [65]. Similar interest in repellents as one of the forms of tick prophylaxis was declared by Slovak students [65].

Results of numerous studies indicate that preventive behaviors are related to the socio-demographic characteristics of respondents, including the sex, age, education level, economic conditions, and living environment e.g., [59,64,65,66,67,68,69,70,71]. The students were not asked about their standard of living and the financial status of their families in the survey. Due to the differences in the modes of data acquisition and the demographic diversity of respondent groups, it is difficult to compare the preventive measures and behaviors reported from different parts of the world. In some regions, e.g., in the Neuchâtel canton (Switzerland) and Montérégie in the province of Québec (Canada), the application of tick repellents was reported by 29% and 15% of the total population, respectively, the use of protective clothing was indicated by 53% and 22%, respectively, and the avoidance of wooded areas during high-risk periods was declared by 25% and 15% [68]. The use of repellents is a preventive practice of 21% of Finnish residents visiting tick-infested areas, and 81% of residents wear long sleeves and trousers to protect themselves against ticks [72]. In turn, the most frequent method for protection against tick bites used by the residents of Nantucket Island (Massachusetts) is based on inspection of the body (80%), wearing protective clothes (53%), avoidance of tick areas (34%), and use of tick repellents (11%) [66]. The TBE vaccination rate among respondents in endemic areas in Finland is 40%, and as many as 88% regard TBE vaccination as the best preventive practice [72]. The risk of the indirect and direct effects of tick parasitism can be reduced by rapid removal of attached ticks with various methods [73,74]. The sooner the tick is removed from the skin, the less likely it is to transmit various pathogen species to the host [75]. Some of them are introduced with saliva shortly after the beginning of blood ingestion, e.g., Powassan virus after 15–30 min [76] and *Rickettsia rickettsii* after 2–94 h [77]; other pathogens like *Borrelia burgdorferi* spirochetes are introduced after a period longer than 24–48 h [78,79].

Despite the high risk to human health posed by ticks and pathogen transmission in Germany e.g., [43,80,81,82,83] and Thailand e.g., [45,49,51,53,84,85], the preventive measures recommended by the Centers for Disease Control and Prevention [86] are still infrequently adopted by young the respondents. The high acceptance of personal protection methods expressed by the Polish students (only 7.1% do not comply with the principles of tick prophylaxis) may be associated with the extensive information campaigns on ticks and the effects of their parasitism that has been carried out in public and commercial television stations and in electronic media in Poland for several years. Moreover, the modification of the school biology curriculum, i.e., the emphasis on topics related to threats posed by ticks to human health, may also play a significant role. In southeastern Poland, a measurable effect of changes in the residents’ behavior is the significant decline in the percentage of subjects who do not use any preventive method, which ranged from 21% to 22% in 2000–2005 [64,67].

The present study shows that television and electronic media, besides school education, play an important role in young people’s awareness of health risks posed by ticks. Popular science magazines, family and friends, and medical personnel or other unspecified sources of information were indicated by the respondents less frequently.

The subjective assessment showed large differences in the knowledge of ticks and effects of their parasitism between the three groups of students. The knowledge is still insufficient, especially among the German and Thai students, which indicates that it should be popularized in the mass media and health education should be expanded at all levels. The level of knowledge of the Polish, German, and Thai students may also be influenced by other sociological, psychological, and cultural factors that were not analyzed in the present study. As in this study, it has been shown that the level of knowledge of ticks and tick-borne diseases in other regions of Europe [71,87,88,89] and the world [69,90,91,92,93] is not satisfactory, even among medical personnel [94] and veterinary medicine students [95].

The data on the site of tick attachment to the body provided by the Polish and German students are consistent with other observations where tick specimens were usually attached to lower and upper limbs and the abdomen [41,96]. Interestingly, 70% of the Asian students reported tick attachment on the head and neck. There are no phytosociological data on the tick-infested areas in Thailand where the respondents resided, and there is no information on the outdoor occupational activities performed by these students; hence, this phenomenon is difficult to elucidate. Our other observations have demonstrated that hungry ticks wait for the host on even 1.5-m high plants (unpublished data). They cling to human clothes or animal fur in physical contact with plants, and start feeding after finding a favorable area on the host’s skin.

The risk of tick attacks can be reduced by landscape management and by limitation of the impact of anthropogenic factors, which contribute to extension of the activity seasons, increased tick population size, and presence of tick-borne pathogens in urban and suburban areas [48,97,98].

## 5. Conclusions

The tick preventive behavior declared by young people and the use of recommended methods of tick prevention vary in different regions of the world. The acceptance of the methods of personal protection against tick bites by the inhabitants of these regions is associated with the country and place of residence (town or village), and the level of knowledge about ticks and tick-borne diseases. 

The poor perception of preventive measures and practices and the low level of knowledge among medical students in some regions require development of a new public health education strategy and changes in the education of future medical staff.

## Figures and Tables

**Table 1:** Demographic data of the Polish, German and Thai students.

Demographic Charakter	Total N/%	Polish Students (Group 1) N/%	German Students (Group 2) N/%	Thai Students (Group 3) N/%
Sex *: F + M	636/100	99/15.6	453/71.2	84/13.2
Female	422/66.5	69/69.7	313/69.1	40/48.2
Male	208/32.8	29/29.3	138/30.5	41/49.4
No response	5/0.8	1/1.0	2/0.4	3/3.6
Place of residence *:	635/100	-	-	-
Town	449/70.7	73.73.7	359/79.2	17/20.2
Villige	105/16.5	23/23.2	81/17.9	1/1.2
No response	0/0	3/3.0	13/2.9	66/78.6
Year of study *:	636/100	99/15.6	453/71.2	84/13.2
1st	96/15.1	24/24.2	51/11.3	21/25.0
2nd	67/10.5	1/1.0	58/19.4	8/9.5
3rd	117/18.4	69/69.7	45/9.9	3/3.6
4th	122/19.2	0/0	116/25.6	6/7.1
5th	37/5.8	0/0	37/8.2	0/0
6th	14/2.2	0/0	12/2.6	2/2.4
No response	154/24.2	5/5.1	104/23.0	44/52.4

* single choice questions; N—number of respondents that answered the question; F—female; M—male.

**Table 2:** Methods for tick prophylaxis employed by the Polish, German, and Thai students and their motivations.

Prophylaxis Methods and Motivations for Their Use	Total N/%	Polish Students (Group 1) N/%	German Students (Group 2) N/%	Thai Students (Group 3) N/%	Statistical Analysis
Prophylaxis methods **:					Chi^2^ = 85.355; *p* < 0.001
Repellents	120/19.4	30/30.3	144/32.3	41/54.7	
Protective clothes	264/42.6	79/79.8	144/32.3	41/54.7	
Preventive behavior in tick habitats	127/20.5	37/37.4	58/13.0	32/42.7	
Inspection of the body on return home	391/63.1	86/86.9	283/63.5	22/29.3	
Other methods	42/6.8	3/3.0	32/7.2	7/9.3	
No prophylaxis measures	124/20	7/7.1	100/22.4	17/22.7	
Total	620/100	99/100	446/100	75/100	
Motivations for application of prophylaxis methods *:					Not tested
Risk of tick-borne diseases		83/83.8	152/33.6	5/6.0	
Fear		8/8.1	12/2.6	2/2.4	
Ease of prophylaxis		4/4.0	74/16.3	6/7.1	
Other		1/1.0	0/0	2/2.4	
No response		3/3.0	215/47.5	69/82.1	
Total		99/100	453/100	84/100	

* single choice questions; ** multiple choice questions; N—number of respondents that answered the question.

**Table 3:** Prophylaxis methods used by the females and males among Polish, German, and Thai students.

Prophylaxis Methods Used by the Females and Males **	Total N/%		Polish Students N/%		German Students N/%		Thai Students N/%	
	F	M	F	M	F	M	F	M
Repellents	77/18.6	40/19.9	19/27.5	11/37.9	54/17.4	23/17.2	4/11.1	6/15.8
Protective clothes	181/43.6	82/40.8	56/81.2	22/75.9	107/34.5	37/27.6	18/50.0	23/60.5
Preventive behawior in tick habitats	82/19.8	43/21.4	27/39.1	10/34.5	39/12.6	18/13.4	16/44.4	15/39.5
Inspection of the body on return home	275/66.3	114/56.7	61/88.4	24/82.8	205/66.1	77/57.5	9/25.0	13/34.2
Other methods	25/6.0	16/8.0	1/1.4	2/6.9	21/6.8	11/8.2	3/8.3	3/7.9
No prophylaxis measures	78/18.8	46/22.9	4/5.8	3/10.3	64/20.6	36/26.9	10/27.8	7/18.4
Statistical analysis	chi^2^ = 4.344 *p* = 0.501		chi^2^ = 3.508 *p* = 0.622		chi^2^ = 4.171 *p* = 0.525		chi^2^ = 1.919 *p* = 0.860	

** multiple choice questions; N—number of respondents that answered the question; F—female; M—male.

**Table 4:** Knowledge of health threats posed by ticks declared by the Polish, German, and Thai students and sources of knowledge.

State of Knowledge and Sources of Information	Total N/%	Polish Students (Group 1) N/%	German Students (Group 2) N/%	Thai Students (Group 3) N/%	Statistical Analysis
State of knowledge *:					Chi^2^ = 92.137; *p* < 0.001
Sufficient	358/56.2	59/59.6	282/62.3	15/17.9	
Insufficient	70/11.0	4/4.0	36/7.9	30/35.7	
Difficult to assess	174/27.3	31/31.4	114/25.2	29/34.5	
Limited to some aspects of the problem	16/2.5	4/4.0	12/2.6	0/0	
No answer	17/2.7	1/1.0	9/2.0	10/11.9	
Total	636/100	99/100	453/100	84/100	
Sources of information **:					Chi^2^ = 88.838; *p* < 0.001
Primary school education	92/15.1	21/21.2	56/12.8	15/21.1	
Secondary school education	200/32.8	57/57.6	128/29.2	15/21.1	
Higher education	257/42.3	66/66.7	152/34.6	39/54.9	
Television	127/20.8	29/29.3	85/19.4	13/18.3	
Magazines	110/18.1	13/13.1	90/20.5	7/9.9	
Electronic media	256/42.0	26/26.3	34/7.7	8/11.3	
Family/Friends	100/16.4	6/6.1	91/20.7	3/4.2	
Medical staff	17/2.8	1/1.0	16/3.6	0/0	
Other	63/10.4	2/2.0	57/13.0	4/5.6	
Total	609/100	99/100	439/100	71/100	

* single choice questions; ** multiple choice questions; N—number of respondents that answered the question.

**Table 5:** Circumstances and symptoms of tick bites in the Polish, German, and Thai students.

Circumstances and Symptoms of Tick Bites	Total N/%	Polish Students N/%	German Students N/%	Thai Students N/%	Statistical Analysis
Tick bite incident *:	636/100	99/100	453/100	84/100	Chi^2^ = 37.234; *p* < 0.001
Yes	334/52.5	46/46.5	268/59.2	20/23.8	
No	302/47.5	53/53.5	185/40.8	64/76.2	
Circumstances of tick attack **:	334/100	46/100	268/100	20/100	Chi^2^ = 73.347; *p* < 0.001
Occupational exposure	13/3.89	1/2.2	5/1.9	7/35.0	
Recreational exposure	304/91.0	38/82.6	254/94.8	12/60.0	
Other	25/7.5	11/23.9	12/4.5	2/10.0	
Area of tick attack **:	334/100	46/100	268/100	20/100	Chi^2^ = 14.844; *p* < 0.001
Urban	100/29.9	8/17.4	79/29.5	13/65.0	
Suburban	257/76.9	39/84.8	210/78.4	8/40.0	
Time of day of tick attack **:	334/100	46/100	268/100	20/100	Chi^2^ = 69.665; *p* < 0.001
In the morning	36/10.8	3/6.5	31/11.6	2/10.0	
Before noon	33/9.9	13/28.3	18/6.7	2/10.0	
At noon	77/21.5	10/21.7	61/22.8	1/5.0	
After noon	200/59.9	23/50.0	170/63.4	7/35.0	
In the evening	27/8.1	5/10.9	18/6.7	4/20.0	
At night	13/3.9	3/6.5	4/1.5	6/30.0	
Presence of systemic symptoms in subjects attacked by ticks *:	334/100	46/100	268/100	20/100	Chi^2^ = 37.880; *p* < 0.001
Yes	18/5.4	0/0	11/4.1	7/35.0	
No	316/94.6	46/100	257/95.9	13/65.0	
Use of drugs after tick infestation *:	334/100	46/100	268/100	20/100	Chi^2^ = 0.567; *p* = 0.758
Yes	22/6.6	2/4.3	19/7.1	1/5.0	
No	312/93.4	44/95.7	249/92.9	19/95.0	
Frequency of tick attacks *:	334/100	46/100	268/100	20/100	Not tested
Once	161/48.2	26/56.5	127/47.4	8/40.0	
More than once	173/51.8	20/43.5	141/52.6	12/60.0	

* single choice questions; ** multiple choice questions; N—number of respondents that answered the question.

## References

[1] Jongejan F., Uilenberg G. (2005). The global importance of ticks. Parasitology.

[2] De la Fuente J., Estrada-Pena A., Venzal J.M., Kocan K.M., Sonenshine D.E. (2008). Overview: Ticks as vectors of pathogens that cause disease in humans and animals. Front. Biosci..

[3] Moneret-Vautrin D.A., Beaudouin E., Kanny G., Guérin L., Roche J.F. (1998). Anaphylactic shock caused by ticks (*Ixodes ricinus*). J. Allergy Clin. Immunol..

[4] Fernández-Soto P., Dávila I., Laffond E., Lorente F., Encinas-Grandes A., Pérez-Sánchez R. (2001). Tick-bite-induced anaphylaxis in Spain. Ann. Trop. Med. Parasitol..

[5] Castelli E., Caputo V., Morello V., Tomasino R.M. (2008). Local Reactions to Tick Bites. Am. J. Dermatopathol..

[6] Buczek W., Buczek A.M., Bartosik K., Buczek A. (2020). Comparison of Skin Lesions Caused by *Ixodes ricinus* Ticks and *Lipoptena cervi* Deer Keds Infesting Humans in the Natural Environment. Int. J. Environ. Res. Public Health.

[7] Buczek A., Sodowska H., Barańska E., Pabis B., Pabis A. (2000). Toxicoses of ticks (Acari: Ixodida). Wiad. Parazytol..

[8] Edlow J.A., McGillicuddy D.C. (2008). Tick paralysis. Infect. Dis. Clin. N. Am..

[9] Diaz J.H. (2010). A 60-year meta-analysis of tick paralysis in the United States: A predictable, preventable, and often misdiagnosed poisoning. J. Med. Toxicol..

[10] Morshed M., Li L., Lee M.K., Fernando K., Lo T., Wong Q.A. (2017). Retrospective Cohort Study of Tick Paralysis in British Columbia. Vector Borne Zoonotic Dis..

[11] Commins S.P., James H.R., Kelly L.A., Pochan S.L., Workman L.J., Perzanowski M.S., Kocan K.M., Fahy J.V., Nganga L.W., Ronmark E. (2011). The relevance of tick bites to the production of IgE antibodies to the mammalian oligosaccharide galactose-α-1,3-galactose. J. Allergy Clin. Immunol..

[12] Van Nunen S.A., O’Connor K.S., Clarke L.R., Boyle R.X., Fernando S.L. (2009). An association between tick bite reactions and red meat allergy in humans. Med. J. Aust..

[13] Van Nunen S. (2015). Tick-induced allergies: Mammalian meat allergy, tick anaphylaxis and their significance. Asia Pac. Allergy.

[14] Villalta D., Pantarotto L., Da Re M., Conte M., Sjolander S., Borres M.P. (2016). High prevalence of SIgE to galactose-α-1,3-galactose in rural pre-alps area: A cross-sectional study. Clin. Exp. Allergy.

[15] Bircher A.J., Hofmeier K.S., Link S., Heijnen I. (2017). Food allergy to the carbohydrate galactose-alpha-1,3-galactose (alpha-gal): Four case reports and a review. Eur. J. Dermatol..

[16] Parola P., Socolovschi C., Jeanjean L., Bitam I., Fournier P.-E., Sotto A., Labauge P., Raoult D. (2008). Warmer Weather Linked to Tick Attack and Emergence of Severe Rickettsioses. PLoS Negl. Trop. Dis..

[17] Süss J., Klaus C., Gerstengarbe F.-W., Werner P.C. (2008). What Makes Ticks Tick? Climate Change, Ticks and Tick-Borne Diseases. J. Trav. Med..

[18] Jaenson T.G., Jaenson D.G.E., Eisen L., Petersson E., Lindgren E. (2012). Changes in the geographical distribution and abundance of the tick *Ixodes ricinus* during the past 30 years in Sweden. Parasit. Vectors.

[19] Jahfari S., Hofhuis A., Fonville M., van der Giessen J., van Pelt W., Sprong H. (2016). Molecular Detection of Tick-Borne Pathogens in Humans with Tick Bites and Erythema Migrans, in the Netherlands. PLoS Negl. Trop. Dis..

[20] Mysterud A., Stigum V.M., Seland I.V., Herland A., Easterday W.R., Jore S., Østerås O., Viljugrein H. (2018). Tick abundance, pathogen prevalence, and disease incidence in two contrasting regions at the northern distribution range of Europe. Parasit. Vectors.

[21] Černý J., Lynn G., Hrnková J., Golovchenko M., Rudenko N., Grubhoffer L. (2020). Management Options for *Ixodes ricinus*-Associated Pathogens: A Review of Prevention Strategies. Int. J. Environ. Res. Public Health.

[22] Daniel M., Benes C., Danielová V., Kríz B. (2011). Sixty years of research of tick-borne encephalitis—A basis of the current knowledge of the epidemiological situation in Central Europe. Epidemiol. Mikrobiol. Imunol..

[23] Süss J. (2011). Tick-borne encephalitis 2010: Epidemiology, risk areas, and virus strains in Europe and Asia—An overview. Ticks Tick Borne Dis..

[24] Maltezou H.C., Papa A. (2011). Crimean-Congo hemorrhagic fever: Epidemiological trends and controversies in treatment. BMC Med..

[25] Stanek G., Wormser G.P., Gray J., Strle F. (2012). Lyme borreliosis. Lancet.

[26] Amicizia D., Domnich A., Panatto D., Lai P.L., Cristina M.L., Avio U., Gasparini R. (2013). Epidemiology of tick-borne encephalitis (TBE) in Europe and its prevention by available vaccines. Hum. Vaccines Immunother..

[27] Centers for Disease Control and Prevention Tick-Borne Relapsing Fever. http://www.cdc.gov/relapsing-fever/.

[28] World Health Organization (WHO) Vector-Borne Diseases. https://www.who.int/mediacentre/factsheets/fs387/en/index10.html.

[29] Petrulionienė A., Radzišauskienė D., Ambrozaitis A., Čaplinskas S., Paulauskas A., Venalis A. (2020). Epidemiology of Lyme Disease in a Highly Endemic European Zone. Medicina.

[30] Parola P., Paddock C.D., Socolovschi C., Labruna M.B., Mediannikov O., Kernif T., Abdad M.Y., Stenos J., Bitam I., Fournier P.-E. (2013). Update on tick-borne rickettsioses around the world: A geographic approach. Clin. Microbiol. Rev..

[31] George J.E., Pound J.M., Davey R.B. (2004). Chemical control of ticks on cattle and the resistance of these parasites to acaricides. Parasitology.

[32] Abbas R.Z., Zaman M.A., Colwell D.D., Gillearde J., Iqbal Z. (2014). Acaricide resistance in cattle ticks and approaches to its management: The state of play. Vet. Parasitol..

[33] Ray D.E., Forshaw P.J. (2000). Pyrethroid insecticides: Poisoning syndromes, synergies, and therapy. J. Toxicol. Clin. Toxicol..

[34] Litovitz T.L., Klein-Schwartz W., Rodgers G.C., Cobaugh D.J., Youniss J., Omslaer J.C., May M.E., Woolf A.D., Benson B.E. (2002). 2001 Annual report of the American Association of Poison Control Centers Toxic Exposure Surveillance System. Am. J. Emerg. Med..

[35] Dahlgren L., Johnson R.M., Siegfried B.D., Ellis M.D. (2012). Comparative Toxicity of Acaricides to Honey Bee (Hymenoptera: Apidae) Workers and Queens. J. Econ. Entomol..

[36] De Meneghi D., Stachurski F., Adakal H. (2016). Experiences in Tick Control by Acaricide in the Traditional Cattle Sector in Zambia and Burkina Faso: Possible Environmental and Public Health Implications. Front. Public Health.

[37] Buczek A., Bartosik K., Wiśniowski Ł., Tomasiewicz K. (2013). Changes in population abundance of adult *Dermacentor reticulatus* (Acari: Amblyommidae) in long-term investigations in eastern Poland. Ann. Agric. Environ. Med..

[38] Hasle G. (2013). Transport of ixodid ticks and tick-borne pathogens by migratory birds. Front. Cell Infect. Microbiol..

[39] Buczek A.M., Buczek W., Buczek A., Bartosik K. (2020). The Potential Role of Migratory Birds in the Rapid Spread of Ticks and Tick-Borne Pathogens in the Changing Climatic and Environmental Conditions in Europe. Int. J. Environ. Res. Public Health.

[40] Cisak E., Wójcik-Fatla A., Stojek N., Chmielewska-Badora J., Zwoliński J., Buczek A., Dutkiewicz J. (2006). Prevalence of *Borrelia burgdorferi* genospecies in *Ixodes ricinus* ticks from Lublin region (eastern Poland). Ann. Agric. Environ. Med..

[41] Bartosik K., Sitarz M., Szymańska J., Buczek A. (2011). Tick bites on humans in the agricultural and recreational areas in south-eastern Poland. Ann. Agric. Environ. Med..

[42] Chmielewska-Badora J., Zwoliński J., Cisak E., Wójcik-Fatla A., Buczek A., Dutkiewicz J. (2007). Prevalence of *Anaplasma phagocytophilum* in *Ixodes ricinus* ticks determined by polymerase chain reaction with two pairs of primers detecting 16S rRNA and ankA genes. Ann. Agric. Environ. Med..

[43] Galfsky D., Król N., Pfeffer M., Obiegala A. (2019). Long-term trends of tick-borne pathogens in regard to small mammal and tick populations from Saxony, Germany. Parasit. Vectors.

[44] Petney T.N., Kolonin G.V., Robbins R.G. (2007). Southeast Asian ticks (Acari: Ixodida): A historical perspective. Parasitol. Res..

[45] Cornet J.-P., Demoraes F., Souris M., Kittayapong P., Gonzalez J.-P. (2009). Spatial distribution of ticks in Thailand: A discussion basis for tick-borne virus spread assessment. Int. J. Geo-Inf. Assoc. Geo-Inf. Tech..

[46] Reis C., Cote M., Paul R.E., Bonnet S. (2011). Questing ticks in suburban forest are infected by at least six tick-borne pathogens. Vector Borne Zoonotic Dis..

[47] Rizzoli A., Silaghi C., Obiegala A., Rudolf I., Hubálek Z., Földvári G., Plantard O., Vayssier-Taussat M., Bonnet S., Spitalská E. (2014). *Ixodes ricinus* and Its Transmitted Pathogens in Urban and Peri-Urban Areas in Europe: New Hazards and Relevance for Public Health. Front. Public Health.

[48] Oechslin C.P., Heutschi D., Lenz N., Tischhauser W., Péter O., Rais O., Beuret C.M., Leib S.L., Bankoul S., Ackermann-Gäumann R. (2017). Prevalence of tick-borne pathogens in questing *Ixodes ricinus* ticks in urban and suburban areas of Switzerland. Parasit. Vectors.

[49] Hirunkanokpun S., Kittayapong P., Cornet J.P., Gonzalez J.P. (2003). Molecular evidence for novel tick-associated spotted fever group rickettsiae from Thailand. J. Med. Entomol..

[50] Parola P., Cornet J.P., Sanogo Y.O., Miller R.S., Thien H.V., Gonzalez J.P., Raoult D., Telford S.R., Wongsrichanalai C. (2003). Detection of *Ehrlichia* spp., *Anaplasma* spp. *Rickettsia* spp., and other eubacteria in ticks from the Thai–Myanmar border and Vietnam. J. Clin. Microbiol..

[51] Ahantarig A., Trinachartvanit W., Milne J.R. (2008). Tickborne pathogens and diseases of animals and humans in Thailand. Southeast Asian J. Trop. Med. Public Health.

[52] Foongladda S., Inthawong D., Kositanont U., Gaywee J. (2011). *Rickettsia, Ehrlichia, Anaplasma*, and *Bartonella* in ticks and fleas from dogs and cats in Bangkok. Vector Borne Zoon. Dis..

[53] Malaisri P., Hirunkanokpun S., Baimai V., Trinachartvanit W., Ahantarig A. (2015). Detection of *Rickettsia* and *Anaplasma* from hard ticks in Thailand. J. Vector Ecol..

[54] Eamudomkarn C. (2017). Tick-borne pathogens and their zoonotic potential for human infection In Thailand. Chiang Mai Vet. J..

[55] Mansfield K.L., Johnson N., Phipps L.P., Stephenson J.R., Fooks A.R., Solomon T. (2009). Tick-borne encephalitis virus—A review of an emerging zoonosis. J. Gen. Virol..

[56] European Centre for Disease Prevention and Control Epidemiological Situation of Tick-Borne Encephalitis in the European Union and European Free Trade Association Countries. Stockholm 2012..

[57] Cornet J.P., Kittayapong P., Gonzalez J.P. (2004). Le risque de transmission d’arbovirus par les tiques en Thailande [Risk of arbovirus transmission by ticks in Thailand]. Med. Trop..

[58] WHO Publication (2011). Vaccines against Tick-Borne Encephalitis.

[59] Nejezchlebová H., Kiewra D., Žákovská A., Ovesná P. (2016). Students’ attitudes to tick risks. Ann. Agric. Environ. Med..

[60] Bissinger B.W., Roe R.M. (2010). Tick repellents: Past, present, and future. Pestic. Biochem. Phys..

[61] Cisak E., Wójcik-Fatla A., Zając V., Dutkiewicz J. (2012). Repellents and acaricides as personal protection measures in the prevention of tick-borne diseases. Ann. Agric. Environ. Med..

[62] Kiss T., Cadar D., Spînu M. (2012). Tick prevention at a crossroad: New and renewed solutions. Vet. Parasit..

[63] Eisen L., Dolan M.C. (2016). Evidence for Personal Protective Measures to Reduce Human Contact with Blacklegged Ticks and for Environmentally Based Control Methods to Suppress Host-Seeking Blacklegged Ticks and Reduce Infection with Lyme Disease Spirochetes in Tick Vectors and Rodent Reservoirs. J. Med. Entomol..

[64] Bartosik K., Kubrak T., Olszewski T., Jung M., Buczek A. (2008). Prevention of tick bites and protection against tick-borne diseases in south-eastern Poland. Ann. Agric. Environ. Med..

[65] Pańczuk A., Tokarska-Rodak M., Mikuľáková W., Kendrová L., Magurová D. (2019). Exposure to ticks and undertaking *Lyme borreliosis* prevention activities among students from Poland and Slovakia. Ann. Agric. Environ. Med..

[66] Phillips C.B., Liang M.H., Sangha O., Wright E.A., Fossel A.H., Lew R.A., Fossel K.K., Shadick N.A. (2001). Lyme disease and preventive behaviors in residents of Nantucket Island, Massachusetts. Am. J. Prev. Med..

[67] Buczek A., Bartosik K., Olszewski T., Sałata M., Stepuch M., Buczek A., Błaszak C. (2003). Tickbite preventive behaviours among inhabitants of Lublin Region. Arthropods and Hosts.

[68] Aenishaenslin C., Michel P., Ravel A., Gern L., Milord F., Waaub J.-P., Bélanger D. (2015). Factors associated with preventive behaviors regarding Lyme disease in Canada and Switzerland: A comparative study. BMC Public Health.

[69] Butler A.D., Sedghi T., Petrini J.R., Ahmadi R. (2016). Tick-borne disease preventive practices and perceptions in an endemic area. Ticks Tick Borne Dis..

[70] Dernat S., Johany F. (2019). Tick Bite Risk as a Socio-Spatial Representation—An Exploratory Study in Massif Central, France. Land.

[71] Slunge D., Jore S., Krogfelt K.A., Jepsen M.T., Boman A. (2019). Who is afraid of ticks and tick-borne diseases? Results from a cross-sectional survey in Scandinavia. BMC Public Health.

[72] Zöldi V., Turunen T., Lyytikäinen O., Sane J. (2017). Knowledge, attitudes, and practices regarding ticks and tick-borne diseases, Finland. Ticks Tick Borne Dis..

[73] Coleman N., Coleman S. (2017). Methods of tick removal: A systematic review of the literature. AMJ.

[74] Buczek A.M., Buczek W., Buczek A., Blaszak C. (2020). Can freezing feeding ticks during removal from host skin be an effective method in prevention of tick-borne diseases?. Parasitic and Allergenic Arthropods.

[75] Richards S.L., Langley R., Apperson C.S., Watson E. (2017). Do Tick Attachment Times Vary between Different Tick-Pathogen Systems?. Environments.

[76] Ebel G., Kramer L. (2004). Short report: Duration of tick attachment required for transmission of Powassan virus by deer ticks. Am. J. Trop. Med. Hyg..

[77] Saraiva D.G., Soares H.S., Sores J.F., Labruna M.B. (2014). Feeding period required by *Amblyomma aureolatum* ticks for transmission of *Rickettsia rickettsii* to vertebrate hosts. Emerg. Infect. Dis..

[78] Crippa M., Rais O., Gern L. (2002). Investigations on the mode and dynamics of transmission and infectivity of *Borrelia burgdorferi* sensu stricto and *Borrelia afzelii* in *Ixodes ricinus* ticks. Vector Borne Zoonotic Dis..

[79] Eisen L. (2018). Pathogen transmission in relation to duration of attachment by *Ixodes scapularis* ticks. Ticks Tick Borne Dis..

[80] Süss J., Schrader C., Falk U., Wohank N. (2004). Tick-borne encephalitis (TBE) in Germany—Epidemiological data, development of risk areas and virus prevalence in field-collected ticks and in ticks removed from humans. Int. J. Med. Microb..

[81] Silaghi C., Gilles J., Höhle M., Pradel I., Fingerle V., Just F.T. (2008). Prevalence of spotted fever group rickettsiae in *Ixodes ricinus* (Acari: Ixodidae) in Southern Germany. J. Med. Entomol..

[82] Silaghi C., Hamel D., Thiel C., Pfister K., Pfeffer M. (2011). Spotted fever group rickettsiae in ticks, Germany. Emerg. Infect. Dis..

[83] Hildebrandt A., Franke J., Schmoock G., Pauliks K., Krämer A., Straube E. (2011). Diversity and Coexistence of Tick-Borne Pathogens in Central Germany. J. Med. Entomol..

[84] Sangkasuwan V., Chatyingmongkol T., Sukwit S., Eamsila C., Chuenchitra T., Rodkvamtook W., Jiang J., Richards A.L., Lerdthusnee K., Jones J.W. (2007). Description of the first reported human case of spotted fever group rickettsiosis in urban Bangkok. Am. J. Trop. Med. Hyg..

[85] Temmam S., Chrétien D., Bigot T., Dufour E., Petres S., Desquesnes M., Devillers E., Dumarest M., Yousfi L., Jittapalapong S. (2019). Monitoring Silent Spillovers Before Emergence: A Pilot Study at the Tick/Human Interface in Thailand. Front. Microbiol..

[86] CDC (2017). Centers for Disease Control, Prevention, Preventing Tick-Bites. https://www.cdc.gov/lyme/prev/index.html.

[87] Beaujean D.J., Bults M., van Steenbergen J.E., Voeten H.A. (2013). Study on public perceptions and protective behaviors regarding Lyme disease among the general public in the Netherlands: Implications for prevention programs. BMC Public Health.

[88] Slunge D., Boman A. (2018). Learning to live with ticks? The role of exposure and risk perceptions in protective behaviour against tick-borne diseases. PLoS ONE.

[89] Riccò M., Gualerzi G., Ranzieri S., Ferraro P., Bragazzi N.L. (2020). Knowledge, Attitudes, Practices (KAP) of Italian Occupational Physicians towards Tick Borne Encephalitis. Trop. Med. Infect. Dis..

[90] Heller J., Benito-Garcia E., Maher N., Chibnik L., Maher C., Shadick N. (2010). Behavioral and attitudes survey about Lyme disease among a Brazilian population in the endemic area of Martha’s Vineyard, Massachusetts. J. Immigr. Minor. Health.

[91] Valente S.L., Wemple D., Ramos S., Cashman S.B., Savageau J.A. (2015). Preventive behaviors and knowledge of tick-borne illnesses: Results of a survey from an endemic area. J. Public Health Manag. Pract..

[92] Gupta S., Eggers P., Arana A., Kresse B., Rios K., Brown L., Sampson L., Kploanyi M. (2018). Knowledge and preventive behaviors towards tick-borne diseases in Delaware. Ticks Tick-Borne Dis..

[93] St Pierre S.E., Gould O.N., Lloyd V. (2020). Knowledge and Knowledge Needs about Lyme Disease among Occupational and Recreational Users of the Outdoors. Int. J. Environ. Res. Public. Health.

[94] Koculu S., Oncul A., Onal A., Yesilbag Z., Uzun N. (2015). Evaluation of knowledge of the healthcare personnel working in Giresun province regarding Crimean-Congo hemorrhagic fever before and after educational training. J. Vector Borne Dis..

[95] Khamassi Khbou M., Ayadi O., Al-Hosary A.A., Darghouth M.A., Gharbi M. (2020). Knowledge and perception on ticks and tick-borne diseases among veterinary medicine students from the North African countries of Algeria, Egypt, and Tunisia. Parasite Epidemiol. Control..

[96] Wilhelmsson P., Lindblom P., Fryland L., Nyman D., Jaenson T.G.T., Forsberg P., Lindgren P.-E. (2013). Ixodes ricinus ticks removed from humans in Northern Europe: Seasonal pattern of infestation, attachment sites and duration of feeding. Parasit. Vectors.

[97] Corrain R., Drigo M., Fenati M., Menandro M.L., Mondin A., Pasotto D., Martini M. (2012). Study on Ticks and Tick-Borne Zoonoses in Public Parks in Italy. Zoon. Public Heath.

[98] Buczek A., Ciura D., Bartosik K., Zając Z., Kulisz J. (2014). Threat of attacks of *Ixodes ricinus* ticks (Ixodida: Ixodidae) and Lyme borreliosis within urban heat islands in south-western Poland. Parasit. Vectors.

